# Supplementary material for: Association between Menopausal Hormone Therapy and Frailty: Cross-Sectional Study Using National Survey Data in Korea
Source: Healthcare (Basel). 2022 Oct 24;10(11):2121. doi: 10.3390/healthcare10112121 (PMC9690075; doi:10.3390/healthcare10112121)
Supplement: Supplementary file 1 [file healthcare-10-02121-s001.zip › healthcare-1928010-supplementary.pdf]

S1. The 51-item frailty index

No.	Category	Item	Scoring
1	Self-reported	Ever diagnosed with stroke	0= No; 1=Yes
2		Ever diagnosed with MI	
3		Ever diagnosed with angina pectoris	
4		Currently treating depression	
5		Ever diagnosed with thyroid illness	
6		Currently treating asthma	
7		Ever diagnosed with RA	
8		Ever diagnosed with OA<br>**Clinical examination data were used if available.*	
9		KNHANES VI: Current lumbago; KNHANES V: Recent lumbago continuous for 3 months	
10		Diagnosed with any cancer excluding skin cancer within 5 years	
11		Any current restrictions in daily living/social activities due to illness, physical or mental disorder	
12		Any bed stays almost all-day last month	
13		Self-reported health	0=Very good-good; 0.5=common; 1=Bad-very bad
14		(EQ5D) Difficulty walking	0= No; 0.5=A little; 1=Severe
15		(EQ5D) Difficulty self-management	
16		(EQ5D) Difficulty ADL	
17		(EQ5D) Pain	
18		(EQ5D) Depressive	
19		Low physical activity within 1 week (low 20%; adjusted by age, sex)	0= Not low; 1=Low
20	Body measurement	Low weight	0=Not low; 1=Low (BMI <18.5 kg/m²)
21		Obese	0=Not obese (<23); 0.5=Slightly obese (≥25 and <30); 0.25=Borderline obese (≥23 and <25); 0.75=Obese (≥30 and <35); 1=Most obese (BMI ≥35 kg/m²)
22	Blood pressure measurement	Hypertension	0=No; 1=Yes (SBP≥140 mmHg or DBP≥90 or taking anti-hypertensive)<br>**If measurements were not available, survey data were used, i.e., ever diagnosed*
23		Hypotension	0=No; 1=Yes (SBP<90 mmHg or DBP<60)
24		Irregular heart rate	0=No; 1=Yes
25	Blood test	Hypercholesteremia	0=No; 1= Yes (≥240 mg/dL after fasting 8 or more hours, or taking lipid control medications)
26		High-density lipoprotein (HDL)	0=No; 1=Low (<40 mg/dL after fasting 8 or more hours)
27		Triglyceride	0=No; 1=High (≥200 mg/dL after fasting 12 or more hours)
28		Diabetes	0=No; 1=Yes (Glucose ≥126 mg/dL after fasting 8 or more hours, or diagnosed with diabetes by a doctor or taking glucose-lowering medication or administering insulin)<br>**If measurements were not available, survey data were used, i.e., ever diagnosed*
29		Hemoglobin A1c	0=Not high (≤5.6%); 0.5=Borderline (>5.6% and <6.5%); 1=Uncontrolled (≥6.5%)

			**2007~2010: Measured only in patients currently under diabetes treatment/fasting glucose ≥126mg/d; 2011~2012: All participants age ≥10 years)*
30		ALT	0: ≤ULN; 0.5: >ULN, ≤2x ULN; 1: > 2x ULN (ULN, male: >33 IU/L, female: >25 IU/L)
31		AST	0: <ULN; 0.5: ≥ULN, <2x ULN; 1: ≥2x ULN (ULN, 20 IU/L)
32		Anemia	0=No; 1=Yes (M: Hemoglobin <13 g/dL, F: <12 g/dL)
33		Hematocrit	0=Not low; 1=Low (≤24%)
34		Renal failure	0=Not low; 1=Low (Cockcroft-Gault CrCl <60 mL/min) **If measurements were not available, survey data were used, i.e., currently have~*
35		White blood cell count	0=4~10 thous/uL (inclusive); else 1
36		Red blood cell count	0=M: 4.2~6.3 million cells/uL, F: 4.0~5.4 (inclusive); else 1
37		Platelet	0=150~450 thous/uL (inclusive); else 1
38	Chest x-ray examination	Heart disease, i.e., right cardiomyopathy, hypertrophy, congenital/ acquired heart disease	0= No; 1=Yes
39		Active pulmonary disease except for TB	
40		Others, i.e., pulmonary nodule, lung cancer, mediastinal disease	
41		(Survey) Cough	
42		(Survey) Phlegm	
43		(Survey) Chest pain	
44		(Survey) Difficulty in breathing	
45		(Survey) Weight loss	
46		(Survey) Exhaustion	
47	Other examination	(Survey) Poor oral health	0= Very good-good; 0.5=Common; 1=Bad-very bad
48		(Survey) Problems with chewing	0= Not at all/Not really/Okay; 0.5= Uncomfortable; 1=Very uncomfortable
49		(Survey) Problems with talking	
50		(Survey) Problems with hearing	0=No; 1=Yes
51		Poor vision	0=No; 1=Yes

S2. Balance diagnostics of variables included in the propensity score model

Characteristics (reference)	Before IPTW		After IPTW	
	SMD	Variance ratio	SMD	Variance ratio
Middle school, graduated (did not graduate)	-0.48	1.10	-0.04	1.02
Household income, low [a] (not low)	0.41	0.69	0.04	0.98
National insurance, others [b] (self/company)	0.18	0.43	0.02	0.93
Age at menopause	0.02	0.87	0.05	0.87
Number of pregnancies	-0.16	0.96	-0.02	1.18
Use of oral contraceptives, yes (never)	-0.28	1.37	-0.01	1.02
Comorbidities at baseline [c]				
Hypertension	-0.14	1.48	-0.03	1.11
Dyslipidemia	-0.09	1.61	-0.01	1.03
MI/AP	-0.01	1.13	0.00	0.97
Stroke	-0.02	1.26	0.00	0.99
Diabetes	-0.02	1.11	0.00	1.00
Hepatitis B, C, or cirrhosis	-0.01	1.12	-0.06	1.54
Any cancer excluding skin cancer	-0.08	1.77	-0.02	1.19
Renal failure	-0.01	1.12	-0.05	1.84
Depression	-0.12	1.18	-0.04	1.06
Thyroid illness	-0.08	1.53	-0.01	1.05
Asthma	-0.01	1.03	0.00	0.98
Arthritis	-0.05	1.11	0.00	0.99

IPTW, inverse probability of treatment weighting. SMD, standardized mean difference.

a Equivalized household income, i.e., household income/√(number of household members) in the lowest 25%; quartiles stratified by sex and age group.

b Medical Aid Class 1 or 2, no health insurance, or unknown.

c Ever diagnosed before menopause (controls) or MHT (treatment group): hypertension, dyslipidemia, myocardial infarction (MI), angina pectoris (AP), stroke, diabetes, liver disease (hepatitis B or C, cirrhosis), cancer excluding skin cancer, renal failure, depression, thyroid illness (both hyper- and hypothyroidism), asthma, or arthritis (rheumatoid arthritis and osteoarthritis).

S3. Comparison of the number of comorbidities stratified by age at baseline

Age at baseline	Number of comorbidities at baseline [a]	Control (n=6,779)			Treated (n=1,044)			p
		n	n'	% (SE)	n	n'	% (SE)	
<50	0	2200	1484	64.12 (0.99	190	117	52.56 (3.19)	<0.001
	1	927	609	27.65 (0.93)	119	79	33.40 (2.97)	
	≥2	262	163	8.23 (0.60)	47	31	14.03 (2.22)	
50-55	0	1551	944	55.45 (1.18)	209	144	45.49 (2.81)	
	1	844	622	30.84 (1.04)	152	107	34.44 (2.67)	
	≥2	352	230	13.71 (0.82)	82	48	20.07 (2.42)	
55-60	0	309	228	51.00 (2.42)	67	57	37.88 (4.60)	
	1	179	144	30.06 (2.09)	68	56	38.51 (4.40)	
	≥2	119	88	18.94 (1.90)	36	28	23.61 (3.96)	
≥60	0	18	12	49.26 (9.40)	25	19	31.93 (6.59)	
	1	8	6	21.99 (7.63)	25	23	35.92 (6.59)	
	≥2	10	7	28.74 (9.21)	24	23	32.16 (6.24)	

Inverse probability of treatment weighting was not applied. n, unweighted frequency; n', effective sample size (=n/design effect); %, weighted percentage; SE, standard error.

a Ever diagnosed before menopause (controls) or MHT (treatment group): hypertension, dyslipidemia, myocardial infarction (MI), angina pectoris (AP), stroke, diabetes, liver disease (hepatitis B or C, cirrhosis), cancer excluding skin cancer, renal failure, depression, thyroid illness (both hyper- and hypothyroidism), asthma, or arthritis (rheumatoid arthritis and osteoarthritis).

S4. Comparison of characteristics between treatment groups before and after IPTW

Characteristics	Before IPTW							After IPTW						
	Control (n=6,779)			Treated (n=1,044)			p	Control (n=6,779)			Treated (n=1,044)			p
	n	n'	% (SE)	n	n'	% (SE)		n	n'	% (SE)	n	n'	% (SE)	
Frailty index*	0.15 (0.001)			0.13 (0.003)			**<.001**	0.15 (0.001)			0.14 (0.003)			**<.001**
Age at survey*	63.13 (0.17)			58.45 (0.25)			**<.001**	62.66 (0.17)			59.50 (0.32)			**<.001**
Survey year, 2008-2009	2555	1302	34.90 (0.83)	291	166	30.05 (1.89)	**0.016**	2555	1475	34.72 (0.82)	291	42	29.47 (2.11)	**0.020**
2010-2012	4224	1981	65.10 (0.83)	753	464	69.95 (1.89)		4224	1406	65.28 (0.82)	753	124	70.53 (2.11)	
Education, <Middle school	4503	2056	62.46 (0.90)	446	278	41.74 (1.93)	**<.001**	4503	1782	59.01 (0.92)	446	60	58.57 (2.10)	0.844
≥Middle school	2276	997	37.54 (0.90)	598	374	58.26 (1.93)		2276	960	40.99 (0.92)	598	135	41.43 (2.10)	
Household income, Not low	4129	2159	65.31 (0.83)	826	529	80.58 (1.45)	**<.001**	4129	1435	67.86 (0.79)	826	153	68.26 (2.36)	0.870
Low [a]	2650	1294	34.69 (0.83)	218	153	19.42 (1.45)		2650	1462	32.14 (0.79)	218	23	31.74 (2.36)	
Living area, MSA [b]	2705	836	43.56 (1.12)	546	355	52.80 (2.07)	**<.001**	2705	990	44.02 (1.10)	546	83	52.78 (2.36)	**<.001**
Other	4074	1267	56.44 (1.12)	498	266	47.20 (2.07)		4074	1344	55.98 (1.10)	498	71	47.22 (2.36)	
Married and living together, Yes	4322	2416	64.99 (0.77)	823	514	78.22 (1.59)	**<.001**	4322	1510	66.40 (0.76)	823	150	75.13 (1.98)	**<.001**
No [c]	2450	1405	35.01 (0.77)	220	143	21.78 (1.59)		2450	1478	33.60 (0.76)	220	31	24.87 (1.98)	
Insurance, Self/company	6345	4027	94.29 (0.37)	1016	633	97.26 (0.60)	**0.001**	6345	1533	94.79 (0.34)	1016	209	94.92 (1.16)	0.911
Others [d]	434	256	5.71 (0.37)	28	20	2.74 (0.60)		434	403	5.21 (0.34)	28	3	5.08 (1.16)	
Smoking history, Never	6206	3668	90.57 (0.48)	975	617	92.08 (1.06)	0.220	6206	1585	90.72 (0.48)	975	195	91.58 (1.29)	0.541
At least once	567	309	9.43 (0.48)	69	42	7.92 (1.06)		567	436	9.28 (0.48)	69	9	8.42 (1.29)	
Number of pregnancies*	4.82 (0.04)			4.42 (0.08)			**<.001**	4.76 (0.04)			4.70 (0.12)			0.589
Birth experience, 0	141	101	2.08 (0.21)	24	16	2.13 (0.55)	**0.009**	141	157	2.09 (0.21)	24	4	1.99 (0.55)	0.850
≥1	6610	3882	97.92 (0.21)	1016	632	97.87 (0.55)		6610	1470	97.91 (0.21)	1016	222	98.01 (0.55)	
Months of OC use*	4.22 (0.25)			6.75 (0.65)			**<.001**	4.58 (0.27)			5.13 (0.56)			0.384
Age at menarche*	15.99 (0.04)			15.70 (0.07)			**<.001**	15.94 (0.04)			15.86 (0.09)			0.383
Age at menopause*	48.69 (0.08)			48.43 (0.17)			0.153	48.71 (0.08)			48.57 (0.19)			0.518
Baseline comorbidities [e], 0	4078	2826	59.38 (0.73)	491	340	46.29 (1.87)	**<.001**	4078	1905	58.13 (0.73)	491	81	49.85 (2.19)	**<.001**
1	1958	1400	29.13 (0.65)	364	258	34.68 (1.72)		1958	1316	29.56 (0.66)	364	58	35.29 (2.04)	
≥2	743	443	11.49 (0.49)	189	121	19.02 (1.46)		743	517	12.32 (0.53)	189	39	14.86 (1.33)	
Hypertension , No	6281	3856	92.09 (0.42)	910	596	86.68 (1.26)	**<.001**	6281	1615	91.42 (0.46)	910	195	89.14 (1.28)	0.067
Yes	498	298	7.91 (0.42)	134	89	13.32 (1.26)		498	374	8.58 (0.46)	134	22	10.86 (1.28)	
Dyslipidemia, No	6598	4034	97.12 (0.26)	992	625	95.59 (0.72)	**0.023**	6598	1523	96.86 (0.28)	992	217	96.79 (0.64)	0.921
Yes	181	112	2.88 (0.26)	52	41	4.41 (0.72)		181	159	3.14 (0.28)	52	11	3.21 (0.64)	
MI, No	9432	7541	99.97 (0.02)	479	381	100.0 (0.00)	0.806	6760	1493	99.69 (0.09)	1042	227	99.78 (0.17)	0.675
Yes	3	3	0.03 (0.02)	0	0	.		19	17	0.31 (0.09)	2	0	0.22 (0.17)	
AP, No	6718	4261	99.00 (0.14)	1031	647	98.75 (0.43)	0.562	6718	1508	99.00 (0.15)	1031	224	98.86 (0.44)	0.756
Yes	61	43	1.00 (0.14)	13	8	1.25 (0.43)		61	64	1.00 (0.15)	13	2	1.14 (0.44)	
Stroke, No	6730	4241	99.35 (0.11)	1033	638	99.16 (0.31)	0.534	6730	1498	99.31 (0.12)	1033	223	99.33 (0.27)	0.968
Yes	49	38	0.65 (0.11)	11	9	0.84 (0.31)		49	54	0.69 (0.12)	11	3	0.67 (0.27)	
Diabetes, No	6598	3980	97.07 (0.28)	1009	627	96.81 (0.64)	0.704	6598	1511	97.04 (0.28)	1009	215	97.37 (0.53)	0.590

Yes	181	100	2.93 (0.28)	35	26	3.19 (0.64)		181	151	2.96 (0.28)	35	9	2.63 (0.53)	
Hep B/C, cirrhosis, No	6704	4225	98.86 (0.16)	1029	637	98.43 (0.46)	0.329	6704	1504	98.82 (1.16)	1029	220	97.72 (0.76)	0.065
Yes	75	52	1.14 (0.16)	15	11	1.57 (0.46)		75	77	1.18 (1.16)	15	2	2.28 (0.76)	
Cancer, No	6682	4314	98.37 (0.20)	1013	622	97.51 (0.53)	0.081	6682	1530	98.17 (0.22)	1013	217	97.92 (0.52)	0.652
Yes	97	58	1.63 (0.20)	31	26	2.49 (0.53)		97	80	1.83 (0.22)	31	7	2.08 (0.52)	
Renal failure, No	6759	4203	99.72 (0.08)	1040	647	99.67 (0.17)	0.774	6759	1490	99.71 (0.08)	1040	229	99.45 (0.36)	0.353
Yes	20	15	0.28 (0.08)	4	5	0.33 (0.17)		20	22	0.29 (0.08)	4	0	0.55 (0.36)	
Depression, No	5542	3642	82.25 (0.57)	779	508	72.95 (1.75)	**<.001**	5542	1700	81.50 (0.60)	779	137	75.62 (1.88)	**0.001**
Yes	1237	798	17.75 (0.57)	265	158	27.05 (1.75)		1237	871	18.50 (0.60)	265	42	24.38 (1.88)	
Thyroid illness, No	6598	4365	96.92 (0.28)	995	636	95.01 (0. 82)	**0.011**	6598	1567	96.70 (0.30)	995	219	95.78 (0.98)	0.330
Yes	181	99	3.08 (0.28)	49	33	4.99 (0. 82)		181	142	3.30 (0.30)	49	6	4.22 (0.98)	
Asthma, No	6584	4113	97.01 (0.28)	1008	645	96.05 (0.76)	0.203	6584	1525	97.01 (0.28)	1008	218	96.37 (0.78)	0.413
Yes	195	104	2.99 (0.28)	36	22	3.95 (0.76)		195	161	2.99 (0.28)	36	6	3.63 (0.78)	
Arthritis, No	5869	4005	86.26 (0.53)	868	575	83.00 (1.40)	**0.017**	5869	1678	85.95 (0.54)	868	168	84.71 (1.47)	0.406
Yes	910	557	13.74 (0.53)	176	115	17.00 (1.40)		910	674	14.05 (0.54)	176	31	15.29 (1.47)	

Inverse probability of treatment weighting (IPTW) was not applied. * mean (SE).

n, unweighted frequency; *n'*, effective sample size (=*n*/design effect); %, weighted percentage; SE, standard error; OC, oral contraceptives.

a Equivalized household income, i.e., household income/√(# of household members) in the lowest 25%; quartiles stratified by sex and age group.

b Metropolitan statistical areas, i.e., top eight major cities in the Republic of Korea.

c Never married, separated, widowed, or divorced.

d Medical Aid Class 1 or 2, no health insurance, or unknown.

e Ever diagnosed before menopause (controls) or MHT (treatment group): hypertension, dyslipidemia, myocardial infarction (MI), angina pectoris (AP), stroke, diabetes, liver disease (hepatitis B or C, cirrhosis), cancer excluding skin cancer, renal failure, depression, thyroid illness (both hyper- and hypothyroidism), asthma, or arthritis (rheumatoid arthritis and osteoarthritis).

S5. Characteristics associated with frailty (IPTW regression analysis, Model 3)

Characteristics	Exposure: Duration of MHT		Exposure: Age at first MHT		Exposure: Time to MHT initiation	
	**B (SE)**	***p***	**B (SE)**	***p***	**B (SE)**	***p***
Age at survey	0.003 (0.000)	**<.001**	0.003 (0.000)	**<.001**	0.003 (0.000)	**<.001**
Survey year, V (IV)	-0.011 (0.003)	**0.001**	-0.010 (0.003)	**0.001**	-0.011 (0.003)	**<.001**
Education, ≥Middle school (<Middle school)	-0.015 (0.003)	**<.001**	-0.015 (0.003)	**<.001**	-0.015 (0.003)	**<.001**
Household income, Low [a] (Not low)	0.011 (0.004)	**0.002**	0.012 (0.003)	**0.001**	0.011 (0.004)	**0.002**
Living area, Other (MSA [b])	0.009 (0.003)	**0.001**	0.008 (0.003)	**0.003**	0.009 (0.003)	**0.003**
Married and living together, No [c] (Yes)	0.005 (0.003)	0.124	0.005 (0.003)	0.106	0.005 (0.003)	0.097
Insurance, Others [d] (Self/company)	0.028 (0.007)	**<.001**	0.028 (0.007)	**<.001**	0.029 (0.007)	**<.001**
Smoking history, At least once (Never)	0.006 (0.005)	0.218	0.005 (0.005)	0.271	0.005 (0.005)	0.289
Number of pregnancies	0.002 (0.001)	**<.001**	0.002 (0.001)	**<.001**	0.002 (0.001)	**0.001**
Age at menarche	-0.001 (0.001)	0.169	-0.001 (0.001)	0.166	-0.001 (0.001)	0.194
Age at menopause	-0.002 (0.000)	**<.001**	-0.001 (0.000)	**<.001**	-0.002 (0.000)	**<.001**
Baseline comorbidities [e], 1 (0)	0.023 (0.003)	**<.001**	0.022 (0.003)	**<.001**	0.023 (0.003)	**<.001**
≥2 (0)	0.065 (0.005)	**<.001**	0.065 (0.005)	**<.001**	0.066 (0.004)	**<.001**
Baseline depression, Yes (No)	-0.005 (0.004)	0.164	-0.005 (0.004)	0.178	-0.006 (0.004)	0.136

Inverse probability of treatment weighting (IPTW) was applied. SE, standard error.

a Equivalized household income, i.e., household income/√(number of household members) in the lowest 25%; quartiles stratified by sex and age group.

b Metropolitan statistical areas, i.e., top eight major cities in the Republic of Korea.

c Never married, separated, widowed, or divorced.

d Medical Aid Class 1 or 2, no health insurance, or unknown.

e Ever diagnosed before menopause (controls) or MHT (treatment group): hypertension, dyslipidemia, myocardial infarction (MI), angina pectoris (AP), stroke, diabetes, liver disease (hepatitis B or C, cirrhosis), cancer excluding skin cancer, renal failure, depression, thyroid illness (both hyper- and hypothyroidism), asthma, or arthritis (rheumatoid arthritis and osteoarthritis).

S6. Characteristics of the subgroups in KNHANES

**Characteristics**	**Years since menopause [1, 30] (*n* = 6,634)**			**Age at menopause > 45 (*n* = 6,111)**			**No hysterectomy[a] (*n* = 2,653)**			**No bilateral oophorectomy[b] (*n* = 4,746)**		
	n	***n′***	**% (SE)**	***n***	***n′***	**% (SE)**	***n***	***n′***	**% (SE)**	***n***	***n′***	**% (SE)**
Age at survey (years)												
Mean (SE)	61.30 (0.14)			62.17 (0.15)			62.94 (0.26)			62.64 (0.20)		
Min-Max	32-80			46-80			35-80			34-80		
Age at menopause (years)												
Mean (SE)	49.33 (0.07)			50.73 (0.05)			49.00 (0.10)			48.88 (0.09)		
Min-Max	30-62			46-62			30-62			30-62		
Time since menopause (years)												
<10	2594	1475	46.41 (0.81)	2602	1494	50.11 (0.83)	918	470	42.81 (1.34)	1794	929	44.51 (1.00)
10-19	2253	1502	30.82 (0.69)	1925	1369	27.65 (0.68)	768	563	26.85 (1.02)	1341	889	25.28 (0.78)
20-29	1787	1271	22.78 (0.61)	1301	973	18.29 (0.58)	639	485	19.77 (0.88)	1051	777	19.33 (0.67)
≥30	-	-	-	260	198	3.95 (0.29)	327	256	10.57 (0.67)	538	351	10.88 (0.56)
Frailty index (FI)												
Mean (SE)	0.14 (0.00)			0.15 (0.00)			0.16 (0.00)			0.15 (0.00)		
Min-Max	0-0.52			0-0.52			0-0.52			0-0.49		
MHT												
Control	5634	3286	85.10 (0.55)	5285	3329	86.66 (0.55)	2401	1414	88.92 (0.80)	4066	2465	86.57 (0.63)
Treated	1000	774	14.90 (0.55)	826	512	13.34 (0.55)	252	146	11.08 (0.80)	680	415	13.43 (0.63)
Duration of treatment (months)												
Median (IQR)	17.08 (3.24-46.02)			12.00 (3.14-35.09)			11.35 (4.52-34.95)			16.90 (2.74-35.20)		
Min-Max	1-252			1-192			1-204			1-204		
<6 months	286	197	28.85 (1.70)	245	175	29.55 (1.85)	75	47	26.60 (3.18)	202	142	30.73 (2.90)
6 months-2 years	325	197	33.92 (1.88)	276	173	34.66 (2.04)	89	51	38.07 (3.61)	222	144	33.75 (2.25)
>2, ≤5 years	245	156	24.37 (1.72)	206	132	25.66 (1.89)	62	46	25.11 (3.10)	169	103	25.00 (2.19)
>5, ≤8 years	72	54	7.00 (0.95)	52	51	5.32 (0.80)	15	9	6.27 (1.76)	43	44	5.40 (0.90)
>8 years	72	65	5.85 (0.79)	47	37	4.81 (0.84)	11	9	3.95 (1.25)	44	38	5.12 (0.93)
Age at first treatment (years)												
Mean (SE)	50.83 (0.20)			52.09 (0.17)			51.13 (0.36)			51.12 (0.26)		
Min-Max	31-70			42-70			35-69			31-69		
<50	341	265	36.88 (1.92)	193	128	24.48 (2.14)	80	52	31.14 (3.56)	206	119	33.00 (2.74)
≥50, <55	419	333	43.70 (1.94)	408	256	51.72 (2.47)	117	74	48.24 (4.18)	295	185	43.54 (2.80)
≥55, <60	170	163	13.79 (1.27)	161	130	16.79 (1.75)	33	24	10.76 (2.59)	132	104	17.44 (2.02)
≥60	70	81	5.64 (0.76)	64	56	7.00 (1.23)	22	15	9.85 (2.78)	47	47	6.01 (1.18)
Time to treatment initiation after menopause (years)												
Median (IQR)	0.41 (-0.56-2.97)			0.16 (-0.65-2.22)			0.27 (-0.77-2.85)			0.41 (-0.54-2.97)		

Min-Max	-5-23			-5-22			-5-20			-5-27		
Before menopause	99	69	10.43 (1.16)	101	74	13.00 (1.37)	42	26	18.58 (2.89)	61	49	8.81 (1.24)
<1 year	322	199	33.30 (1.83)	273	172	34.39 (2.01)	68	40	27.52 (3.33)	226	136	35.01 (2.34)
1-2 years	236	155	24.92 (1.70)	204	141	26.16 (1.83)	63	41	25.68 (3.21)	152	96	23.78 (2.07)
3-6 years	191	131	18.62 (1.49)	157	122	17.73 (1.50)	41	29	15.72 (2.43)	136	94	19.49 (1.88)
7-10 years	89	75	7.42 (0.91)	52	44	4.86 (0.81)	22	15	8.05 (1.86)	60	55	7.01 (1.07)
≥11 years	63	60	5.31 (0.73)	39	38	3.86 (0.68)	16	17	4.45 (1.15)	45	41	5.91 (0.99)

Inverse probability of treatment weighting was not applied. *n*, unweighted frequency; *n'*, effective sample size (=n/design effect); %, weighted percentage; SE, standard error; IQR, interquartile range.

a Data on hysterectomies were collected in 2008 and 2009 only.

b Data on oophorectomies were collected in 2010, 2011, and 2012 only.
